# Acute necrotizing colitis due to sigmoid colon cancer

**DOI:** 10.1186/1477-7819-12-19

**Published:** 2014-01-27

**Authors:** Hiroki Matsunaga, Dai Shida, Makoto Kamesaki, Yuichi Hamabe

**Affiliations:** 1 Department of Surgery, Tokyo Metropolitan Bokutoh Hospital, 4-23-15 Koto-bashi, Sumida-ku, Tokyo 1308575, Japan; 2 Tertiary Emergency Medical Center, Tokyo Metropolitan Bokutoh Hospital, 4-23-15 Koto-bashi, Sumida-ku, Tokyo 1308575, Japan; 3 Colorectal Surgery Division, National Cancer Center Hospital, 5-1-1 Tsukiji, Chuo-ku, Tokyo 1040045, Japan

**Keywords:** Necrotizing colitis, Fulminant obstructive colitis, Obstructed colon cancer

## Abstract

When obstructive colitis becomes fulminant, it is known as ‘acute necrotizing colitis’. We report a rare case of acute necrotizing colitis due to sigmoid colon cancer, in which shock status occurred within ten hours of onset. A 79-year-old female with acute abdominal pain was transported to our hospital with acute shock. Abdominal enhanced computed tomography revealed thickening of the wall of the sigmoid colon and marked dilation of the proximal colon. Emergency surgery was performed with the intraoperative findings of severe sigmoid colon stenosis and proximal dilation of the colon without perforation, and a large volume of putrid ascitic fluid. The intestine was proximally dilated and black in color, from the sigmoid colon to the ileum 60 cm proximal to the terminal ileum, suggesting acute necrosis. Total colectomy with 80 cm resection of terminal ileum and ileostomy was performed. Whereas acute necrotizing colitis is a rare condition and its etiology remains obscure, the chronic ischemic state must play some role. Our patient was of advanced age and had diabetes mellitus and hypertension. These factors might lead to a chronic ischemic state of the bowel due to arteriosclerosis. In addition to the underlying condition, massive bacterial reflux into the ileum from the colon might cause the capillary vasoconstriction of the bowel that led to her critical state.

## Background

Obstructive colitis is a condition defined as nonspecific inflammatory lesions of the colon, such as erosion and ulceration, proximal to a completely or partially obstructive lesion [1-3]. Obstructive colitis, although rare, can become fulminant, which is then known as ‘acute necrotizing colitis’ [4,5]. Extensive colonic gangrene associated with colonic obstruction is a serious complication requiring emergency surgery. Only few cases of acute necrotizing colitis due to colon cancer are reported in the English medical literature [6-8].

We experienced a case of acute necrotizing colitis and ileitis due to colon cancer with a rapid clinical course, in which shock status occurred within ten hour of initial symptoms. The patient was saved only after intensive care and two emergency operations, thus emphasizing the importance of these interventions in such cases.

## Case presentation

**Figure 1:**
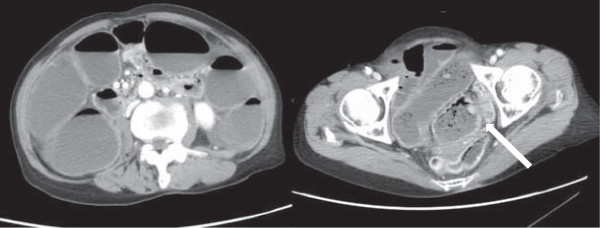
Abdominal enhanced computed tomography scan showing a large tumor in the sigmoid colon (arrow) and dilation of the colon and small intestine.

**Figure 2:**
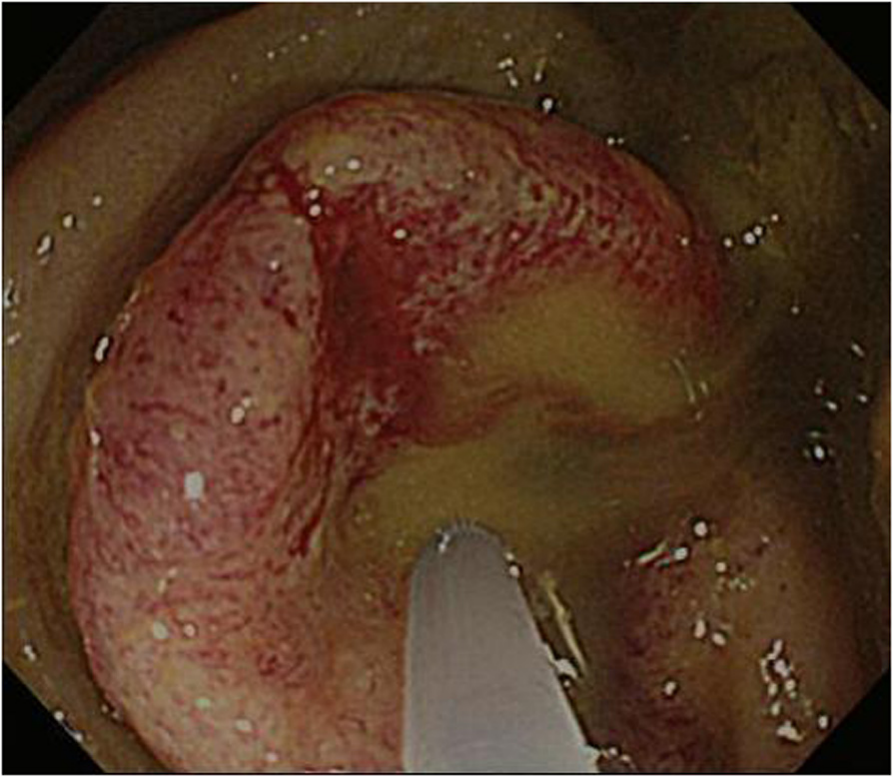
Colonoscopy showing an obstructive tumor in the sigmoid colon.

**Figure 3:**
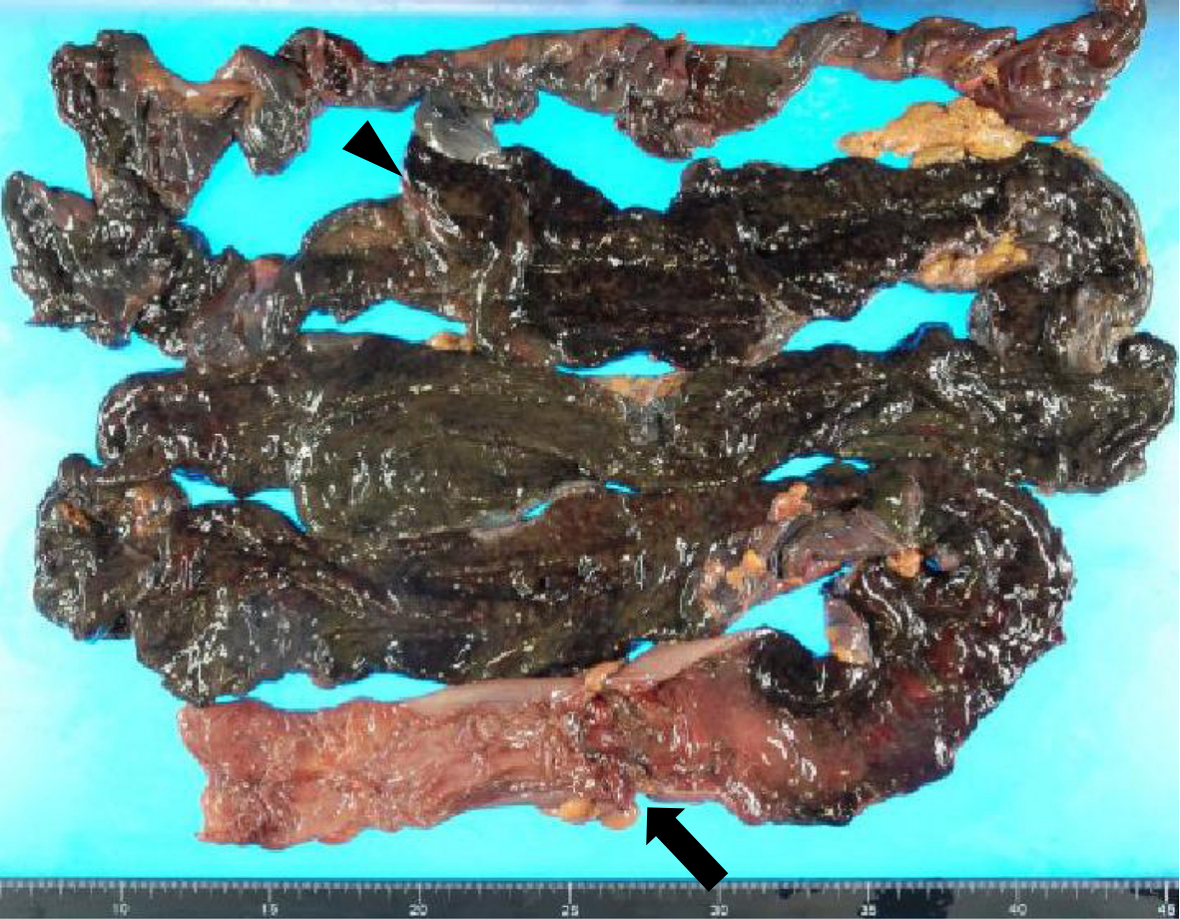
**Macroscopic appearance of the resected intestine.** Arrow indicates colon cancer, and arrow head indicates the ileocecal valve.

A 79-year-old female with acute, gradually worsening abdominal pain requested emergency services. By the time of their arrival, she had collapsed. She was transported to our tertiary emergency medical center. Physical examination on admission revealed a Glasgow coma scale of E4V5M6, blood pressure 80/50 mmHg, pulse 88/min, respiratory rate 28/minute, temperature 36.9°C, and O_2_ saturation 100% (with 10 l reservoir mask). Acute shock, abdominal distension and tenderness, and muscular guarding were also noted. Laboratory results showed a mild increase in the inflammatory response: leukocyte count 7.6 × 10^3^/μl, C-reactive protein 2.70 mg/dl, hemoglobin 15.3 mg/dl, and blood urea nitrogen 41 mg/dl, indicative of severe dehydration. Abdominal enhanced computed tomography (CT) revealed thickening of the sigmoid colon and marked dilation of the proximal intestine from the ileum to the sigmoid colon (Figure 1). Emergency colonoscopy showed an advanced colorectal carcinoma with severe and complete circumferential stenosis (Figure 2). Respiratory status, consciousness, and circulatory status deteriorated gradually. Blood gas analysis showed pH 6.967, PaO_2_ 48.6 mmHg, and PaCO_2_ 77.0 mmHg, indicating ventilatory failure. We thus performed emergency laparotomy under a diagnosis of fulminant obstructive colitis due to sigmoid colon cancer. Intraoperative findings were severe stenosis of the sigmoid colon and proximal dilation without perforation, and a large volume of putrid ascitic fluid. The intestine was dilated proximally and colored black from the sigmoid colon to 60 cm proximal to the terminal ileum, suggesting acute necrosis without major vessel occlusion. We performed total colectomy with 80 cm resection of terminal ileum and ileostomy in an operating time of two and a half hours. Macroscopic findings showed a tumor (45 × 35 mm) in the sigmoid colon causing colonic obstruction. The bowel, from the sigmoid colon to 60 cm proximal to the terminal ileum, was totally necrotized (Figure 3). Normal mucosa was observed between the necrotic lesion and the tumor in the sigmoid colon, which was compatible with a diagnosis of obstructive colitis (Figure 3). Histopathologic diagnosis was moderately differentiated adenocarcinoma and was evaluated as T3N0M0 stage II according to the American Joint Committee on Cancer (AJCC) staging system.

After surgery, intensive care, including mechanical ventilation, continuous hemodiafiltration, polymyxin B hemoperfusion, and blood transfusion, was continued to address circulatory failure and acute renal failure due to sepsis. The patient’s general condition improved gradually, and catecholamine administration was withdrawn on postoperative day 5. On postoperative day 8, perforation of the small intestine near the stoma occurred. We performed a further emergency surgery to create a new ileostomy. The patient’s general condition improved, and she was then transferred to a rehabilitation hospital on postoperative day 71.

## Conclusions

The incidence of obstructive colitis is known to be 0.3% to 3.1% of all colorectal carcinomas [1,3]; however, it remains an uncommon and troublesome disease. The features of obstructive colitis are as follows: (1) an ulcero-inflammatory lesion present on the oral side of the obstruction site; (2) the anal side of the obstruction site is macroscopically and histologically normal; and (3) normal mucosa is present between the obstruction and ulceration sites, and its border is clear [2]. The findings in our case were according to these criteria.

Among obstructive colitis cases, few develop fulminating gangrene of the colon, an entity is termed ‘necrotizing colitis’ [4], the etiology of which remains unknown. Increasing intraluminal pressure may affect colonic blood flow, and in closed-loop colonic obstruction without perforation, mucosal ischemia may be the primary event leading to massive colonic gangrene. Bacterial proliferation in the obstructed fecal material may be a factor [5], and hypoxia may also play a key role. Hypoxia stimulates the germination of spores and bacterial growth, which then progresses rapidly to produce exotoxins that destroy and liquefy surrounding tissue, leading to rapid spread of the disease [5]. Our patient was of advanced age and had diabetes mellitus and hypertension. These factors might lead to chronic ischemic state of the bowel due to arteriosclerosis. In addition to the colitic condition, massive bacterial reflux into the ileum from the colon caused the capillary vasoconstriction of the bowel and that led to her critical condition.

Since the present case followed a rapid clinical course and entered shock status within ten hours, we performed emergency surgery. At the tertiary emergency room, rapid 2 l-infusion was initially administered and at that time, the patient was responding to some extent. So, enhanced CT and colonography were examined. With colonography, a transanal drainage tube was inserted to the proximal dilated bowel through the tumor. Because her vital signs deteriorated in spite of these treatments, we performed the emergency operation. In our case, examination of enhanced CT and colonography did not change the final management, and might simply have delayed the operation. Urgent laparotomy might, therefore, be the better choice in septic shock and hemodynamically unstable conditions with clear peritoneal signs on arrival.

In this situation, it is vital to resect the entire necrotic intestine, following which the status of the mucosa of the proximal margin and proximal remnant bowel should be checked. If any ischemic change is observed in the proximal remnant bowel mucosa, further colonic tissue should be resected proximally until a healthy mucosal margin is achieved [1]. In the present case, during the first operation, we confirmed that mucosal status at the proximal margin was normal. However, on postoperative day 8, the delayed perforation that occurred near this area suggested that acute necrotizing colitis had been progressive from the time of the first operation. Therefore, it would seem advisable to resect more intestine than that indicated solely by macroscopic findings.

In summary, we report a case of acute necrotizing colitis due to sigmoid colon cancer, in which shock status occurred 10 hours from onset. Emergency surgery and intensive care were essential in saving the patient’s life.

## Consent

Written informed consent was obtained from the patient for publication of this case report and any accompanying images. A copy of the written consent is available for review by the Editor-in-Chief of this journal.

## Abbreviations

AJCC: American Joint Committee on Cancer; CT: computed tomography.

## Competing interests

The authors declare that they have no competing interests.

## Author’ contributions

HM, MK and YH collected the data, performed the treatment, and wrote the paper. DS was responsible for writing the paper and for its supervision. All authors read and approved the final manuscript.
